# *THADA*, *SDHAF4*, and *MACF1* Gene Polymorphisms and Placental Expression in Women with Gestational Diabetes

**DOI:** 10.3390/genes14010083

**Published:** 2022-12-27

**Authors:** Przemysław Ustianowski, Damian Malinowski, Michał Czerewaty, Krzysztof Safranow, Maciej Tarnowski, Violetta Dziedziejko, Andrzej Pawlik

**Affiliations:** 1 Department of Nursing, Pomeranian Medical University, 70-210 Szczecin, Poland; 2 Department of Experimental and Clinical Pharmacology, Pomeranian Medical University, 70-111 Szczecin, Poland; 3 Department of Physiology, Pomeranian Medical University, 70-111 Szczecin, Poland; 4 Department of Biochemistry and Medical Chemistry, Pomeranian Medical University, 70-111 Szczecin, Poland; 5 Department of Physiology in Health Sciences, Pomeranian Medical University, 70-210 Szczecin, Poland

**Keywords:** gestational diabetes, polymorphism, placenta

## Abstract

Gestational diabetes mellitus (GDM) is a metabolic disorder in pregnant women leading to various complications. Consequently, factors predisposing its development are being sought. Previous studies have shown that the pathogenesis of GDM is similar to that of type 2 diabetes, and it is therefore thought that the two diseases may have a common genetic basis. The aim of this study was to examine the associations between thyroid adenoma-associated (*THADA*) rs7578597 T>C, succinate dehydrogenase complex assembly factor 4 (*SDHAF4*) rs1048886 A>G, and microtubule-actin crosslinking factor 1 (*MACF1*) rs2296172 A>G gene polymorphisms and the risk of GDM development as well as selected clinical parameters in women with GDM. We also examined the expression of these genes in the placenta of women with and without GDM in association with clinical parameters. This case-control study included 272 pregnant women with GDM and 348 pregnant women with normal glucose tolerance. There were no statistically significant differences in the distribution of the *THADA* rs7578597 T>C, *SDHAF4* rs1048886 A>G, and *MACF1* rs2296172 A>G gene polymorphisms between pregnant control women and women with GDM. The associations between clinical parameters such as body mass before pregnancy, body mass at birth, body mass increase during pregnancy, BMI before pregnancy, BMI at birth, BMI increase during pregnancy, glycated hemoglobin (HbA1c), daily insulin requirement, childbirth time, and newborn body mass and APGAR score, and the *THADA* rs7578597 T>C, *SDHAF4* rs1048886 A>G, and *MACF1* rs2296172 A>G genotypes were statistically non-significant. We only observed lower values of body mass before pregnancy and body mass at birth in women with the *SDHAF4* rs1048886 AG genotype in comparison with AA genotype carriers. There was no statistically significant difference in the expression of *THADA*, *SDHAF4*, and *MACF1* genes in the placenta between women with GDM and healthy women. There were also no statistically significant correlations between *THADA*, *SDHAF4*, and *MACF1* gene expression in the placenta and clinical parameters. The results of our study suggest that *THADA* rs7578597 T>C, *SDHAF4* rs1048886 A>G, and *MACF1* rs2296172 A>G gene polymorphisms are not significant factors associated with GDM onset. In addition, *SDHAF4* rs1048886 A>G may be associated with body mass before pregnancy and body mass at birth in pregnant women.

## 1. Introduction

Gestational diabetes mellitus (GDM, gestational diabetes mellitus) is a disorder of carbohydrate tolerance occurring in pregnant women [1]. GDM is characterized by pancreatic β-cell dysfunction caused by a number of factors. The pathogenesis of GDM is complex and includes risk factors such as age, obesity, and a family history of diabetes. Many different factors lead to the dysfunction of the pancreatic β-cells, resulting in inadequate postprandial insulin secretion [2]. A second reason for impaired carbohydrate metabolism is tissue insulin resistance. One of the causes of impaired pancreatic β-cell function and insulin resistance is the chronic inflammation observed in pregnant women. Increased expression of inflammatory mediators is also found in the placentas of women with GDM [3,4]. Previous studies have shown that the pathogenesis of GDM is similar to that of type 2 diabetes, and it is therefore thought that the two diseases may have a common genetic basis [5,6]. Recent studies have provided new evidence highlighting the role of neoangiogenesis and inflammation in the pathophysiology of gestational diabetes [7,8]. Association studies have identified a number of genes related to pancreatic β-cell development, function, survival, metabolism, and inflammation that influence the risk of T2DM and GDM [9,10]. The role of type 2 diabetes-related genes in the pathogenesis of GDM is currently under investigation [11,12,13]. 

The Thyroid Adenoma Associated (THADA) gene has been identified as one of the genes associated with the risk of developing type 2 diabetes [14]. This gene is considered to play an important role in the body’s adaptation to environmental conditions, especially cold weather. Some studies have shown an effect of this gene on pancreatic β-cell function and thus on carbohydrate metabolism [14]. To date, a number of studies have been published indicating a link between this gene and type 2 diabetes [15,16].

Succinate dehydrogenase (SDH) has a pivotal position in cellular energy production, linking the tricarboxylic cycle to the electron transport chain [17]. Through the regulation of these processes, SDH influences mitochondrial functioning. Previous studies have shown this enzyme plays an important role in modulating the production of reactive oxygen species, aging, cardiovascular disease, and diabetes [18]. Microtubule actin crosslinking factor 1 (MACF1), also known as actin crosslinking factor 7 (ACF7), belongs to the spectraplakin family [19]. Its expression has been found in many cells and tissues, including skeletal muscle, the heart, pituitary gland, thyroid, salivary glands, mammary glands, liver, kidney, and pancreas [19]. It is involved in the regulation of many cellular and metabolic processes and influences embryonic development [19].

Since these genes affect carbohydrate and lipid metabolism and pancreatic function, as well as may be associated with type 2 diabetes, we decided to investigate the association of polymorphisms of these genes with GDM. It has been shown that polymorphisms of these genes may also be associated with type 2 diabetes risk and parameters of carbohydrate metabolism [15].

In this study, we examined the associations between *THADA* rs7578597 T>C, *SDHAF4* rs1048886 A>G, and *MACF1* rs2296172 A>G gene polymorphisms and the risk of GDM development as well as selected clinical parameters in women with GDM. We also assessed the expression of these genes in the placenta of women with and without GDM in association with clinical parameters.

## 2. Materials and Methods

This case-control study included 272 pregnant women with GDM and 348 pregnant women with normal glucose tolerance (NGT) treated in the Department of Obstetrics and Gynecology, Pomeranian Medical University, Szczecin, Poland. GDM was diagnosed on the basis of a 75 g oral glucose tolerance test (OGTT) at 24–28 weeks gestation, according to the International Association of Diabetes and Pregnancy Study Groups (IADPSG) criteria [20]. Blood samples were taken at enrollment. GDM diagnosis criteria were when one of the following plasma glucose values in the OGTT was met or exceeded: fasting plasma glucose 92 mg/dL (5.1 mmol/L), 1 h plasma glucose 180 mg/dL (10.0 mmol/L), or 2 h plasma glucose 153 mg/dL (8.5 mmol/L). A total of 78% of pregnant women with GDM were treated throughout the pregnancy with dietary control alone, while the remaining 22% of them were treated with diet control and insulin until delivery. Insulin therapy was implemented if morning glycemia was greater than 95 mg/dL (5.6 mmol/L) (for three consecutive days despite an adequate diet or glycemia after one of the main meals was greater than 140 mg/dL (7.8 mmol/L). The dose of insulin was adjusted according to serum glucose values by taking a starting dose of 0.7 IU/kg body weight/24 h. The dose was adjusted daily according to blood glucose levels measured four times a day. In the women included in the study, clinical parameters such as fasting glucose, daily insulin requirement, body mass before pregnancy, body mass at birth, body mass increase during pregnancy, BMI before pregnancy, BMI at birth, BMI increase during pregnancy, and newborn body mass and APGAR score were analyzed. The criteria for patient exclusion from the study were: diabetes type 1 and type 2, autoimmune and inflammatory diseases, neoplastic diseases, chronic infections, acute or chronic complications such as diabetic ketoacidosis, or other disorders affecting glucose metabolism. The study was approved by the local Ethics Committee of Pomeranian Medical University, Szczecin, Poland (KB-0012/40/14), and written informed consent was obtained from all subjects.

### 2.1. Methods

All samples were genotyped (in two technical repeats) using allelic discrimination assays (TaqMan^®^ probes, Applied Biosystems, Waltham, MA, USA) on a 7500 Fast Real-Time PCR Detection System (Applied Biosystems, Carlsbad, CA, USA). In order to discriminate the polymorphisms, we employed TaqMan^®^ Pre-Designed SNP Genotyping Assays, including appropriate primers and fluorescently labeled (FAM and VIC) MGB™ probes to detect the alleles.

### 2.2. Determination of THADA rs7578597 T>C, SDHAF4 rs1048886 A>G, and MACF1 rs2296172 A>G Gene Polymorphisms

Genomic DNA was extracted from peripheral blood samples (200 µL) using the GeneMATRIX Quick Blood DNA Purification Kit (EURx, Gdansk, Poland) following the manufacturer’s manual. Genotyping was performed in a final volume of 12 µL. To discriminate *THADA* rs7578597 T>C, *SDHAF4* rs1048886 A>G, and *MACF1* rs2296172 A>G gene polymorphisms, TaqMan^®^ Pre-Designed SNP Genotyping Assays were used (assay IDs: C__32653841_10, C___7494766_10, and C__15751994_10).

### 2.3. Determination of THADA, SDHAF4, and MACF1 Gene Expression in Placenta

#### 2.3.1. RNA Isolation

For the purpose of this study, randomly selected placentas from 29 women with GDM and 29 healthy women (control group) who had a vaginal delivery after 37 weeks of gestation were obtained. All samples were collected at the Department of Obstetrics and Gynecology, Pomeranian Medical University in Szczecin. The whole placenta was placed in 0.9% NaCl and immediately transported to the Department of Physiology for preservation in further studies just after the delivery. The placental samples were resected from the maternal side of the cotyledons, approximately 100 mg tissue samples, for RNA extraction. No visible connective tissue, vessels, or calcium deposits were detected in the studied material. Total RNA was extracted from obtained cell homogenates using the RNeasy Fibrous Tissue Mini Kit (Qiagen, Hilden, Germany) following the manufacturer’s protocol. The concentration and purity of RNA samples were determined by measuring the absorbance at 260nm using a Perkin Elmer Lambda Bio+ spectrophotometer (PerkinElmer Waltham, MA, USA).

#### 2.3.2. Real-Time Quantitative Reverse Transcription PCR (RQ-PCR)

mRNA isolated from each sample (0.4 μg) was reverse transcribed into cDNA (total volume of 20 μL) using a cDNA synthesis kit (RevertAid RT Kit, Thermo Scientific, Waltham, MA, USA) according to the manufacturer’s manual. The expression of *THADA*, *SDHAF4*, and *MACF1* genes as well as the reference gene was performed using real-time PCR on an ABI PRISM^®^ Fast 7500 Sequence Detection System (Applied Biosystems, Carlsbad, CA, USA), as described previously [16]. mRNA normalization between different sample levels was performed using β-2 microglobulin (*BMG*) as the reference gene. The reference gene was determined based on the available and up-to-date literature data [17,18,19]. Samples were analyzed in two technical repeats. Mean cycle threshold (CT) values were used for further analysis. Each reaction (total volume of 20 μL) contained 2 μL of diluted cDNA. The 2^−ΔCt^ method was used to calculate the values.

### 2.4. Statistical Analysis

The genotype distribution consistency was assessed using the exact test with Hardy–Weinberg equilibrium (HWE). A chi-squared test was used to compare the genotype and allele distributions between the analyzed groups. Quantitative variables were compared across all genotype groups using the Mann–Whitney test. A multivariate logistic regression model was used to find independent predictors of GDM risk. *p*-values < 0.05 were considered statistically significant.

## 3. Results

The distributions of the studied polymorphisms were in HWE (*p* > 0.05). The distribution of studied polymorphisms in the total group of pregnant women with normal carbohydrate tolerance and women with GDM is shown in Table 1. As shown in Table 1, there were no statistically significant differences in the distribution of the *THADA* rs7578597 T>C, *SDHAF4* rs1048886 A>G, and *MACF1* rs2296172 A>G gene polymorphisms between pregnant control women and women with GDM.

There were also no statistical differences in the distribution of studied polymorphisms between the group of GDM women treated with insulin and control women (data not shown).

We also examined the associations between the *THADA* rs7578597 T>C, *SDHAF4* rs1048886 A>G, and *MACF1* rs2296172 A>G gene polymorphisms and clinical parameters, such as body mass before pregnancy, body mass at birth, body mass increase during pregnancy, BMI before pregnancy, BMI at birth, BMI increase during pregnancy, glycated hemoglobin (HbA1c), daily insulin requirement, childbirth time, and newborn body mass and APGAR score in women with GDM (Table 2, Table 3 and Table 4). The associations between the above parameters and the *THADA* rs7578597 T>C, *SDHAF4* rs1048886 A>G, and *MACF1* rs2296172 A>G genotypes were statistically non-significant. We only observed lower values of body mass before pregnancy and body mass at birth in women with the *SDHAF4* rs1048886 AG genotype in comparison with AA genotype carriers

The next step of our study was to examine the expression of the *THADA*, *SDHAF4*, and *MACF1* genes in the placenta of women with and without GDM.

The expression of the *THADA* gene in women with and without GDM was 0.003 ± 0.003 and 0.0027 ± 0.0023, respectively. The differences between women with and without GDM were statistically non-significant (*p* = 0.91). The expression of the *SDHAF4* gene in women with and without GDM was 0.023 ± 0.019 and 0.042 ± 0.061, respectively. These differences were not statistically significant (*p* = 0.27). The expression of the *MACF1* gene in women with GDM and with normal glucose tolerance was 0.0022 ± 0.0095 and 0.00038 ± 0.00074, respectively. No statistically significant differences were found (*p* = 0.56).

We also examined correlations between expression in the placenta of *THADA*, *SDHAF4*, and *MACF1* genes in women with GDM and clinical parameters. The correlations between *THADA*, *SDHAF4*, and *MACF1* gene expression in the placenta and clinical parameters were statistically non-significant (Table 5, Table 6 and Table 7).

Additionally, we compared the mRNA levels of studied genes between women with corresponding genotypes (Table 8). As shown in Table 8 these differences were statistically non-significant.

## 4. Discussion

In this study, we examined the associations between the *THADA* rs7578597 T>C, *SDHAF4* rs1048886 A>G, and *MACF1* rs2296172 A>G gene polymorphisms and the risk of GDM development as well as selected clinical parameters in women with GDM. We also examined the expression of these genes in the placenta of women with and without GDM, as well as the association between the expression of these genes in the placenta and clinical parameters. We found no statistically significant differences in the distribution of the studied polymorphisms between women with GDM and pregnant women with normal glucose tolerance, suggesting that these gene polymorphisms are not significant risk factors for an increased risk of GDM development in our population. Additionally, there was not a statistically significant association between the studied polymorphisms and clinical parameters, except for lower values of body mass before pregnancy and body mass at birth in women with the *SDHAF4* rs1048886 AG genotype in comparison with AA genotype carriers. Moreover, we found no differences in *THADA*, *SDHAF4*, and *MACF1* gene expression between GDM women and women with normal glucose tolerance. There were no statistically significant correlations between the expression of these genes in the placenta and clinical parameters.

GDM is a disorder that occurs during pregnancy, causing a wide variety of complications in both women and newborns [2]. Therefore, factors predisposing to this complication are being sought. The factors associated with the risk of developing type 2 diabetes are considered, as well as a number of factors affecting pancreatic β-cell function, including genetic factors [5,6]. Identifying the factors that increase the risk of GDM can help in the early diagnosis of this complication and the early implementation of appropriate prophylaxis.

Previous studies have suggested that the THADA gene may be associated with an increased risk of developing type 2 diabetes [14,15]. However, studies to date have been inconclusive. Results vary between populations. Studies have suggested that THADA may be associated with metabolic syndrome and insulin resistance. Studies have suggested an effect of the THADA gene on insulin secretion and insulin resistance, as well as an effect on body mass index. To date, the association of THADA rs7578597 T>C with diabetes type 2 risk has been assessed, but the results are inconsistent. In the Chinese population, there was no association of THADA rs7578597 T>C with DT2 risk, but the polymorphism was correlated with glycemic values during oral glucose tolerance [24]. Additionally, in the European population, no association was found between THADA rs7578597 T>C and diabetes [15,25,26], but an association between the THADA gene rs7578597 T>C polymorphism with type 2 diabetes was observed in an Indian population [27]. In a Mexican population, rs7578597 T>C and THADA were significantly associated with obesity, glycemic, and lipid phenotypes in patients with type 2 diabetes [28].

To date, the THADA gene rs7578597 T>C polymorphism has not been widely studied in pregnant women. Stuebe et al. found an association between the THADA gene rs7578597 T>C polymorphism and gestational weight gain [29]. The results of our study suggest that THADA rs7578597 T>C as well as the expression of this gene in the placenta does not play an important role in the pathogenesis of GDM.

Succinate dehydrogenase (SDH) plays an important role in mitochondrial respiratory metabolism. The SDH complex consists of four basic subunits and a number of cofactors that must be correctly assembled to ensure enzyme function [18]. The SDHAF4 gene encodes for assembly factors of the succinate dehydrogenase complex, but the understanding of its role in physiological processes and disease is limited. It has been shown that SDHAF4 regulates mitochondrial succinate dehydrogenase activity and thus many metabolic processes in the human organism [30].

Succinate dehydrogenase (SDH) is an enzyme that plays a key role in cellular energy production, linking the tricarboxylic acid cycle to the electron transport chain [30]. Therefore, it is involved in regulating a number of metabolic processes, including carbohydrate metabolism. It has been shown that dysfunction of this enzyme in pancreatic β-cells leads to impaired insulin secretion and diabetes [18]. Dysfunction of the mitochondria of β-cells plays a pivotal role in type 2 diabetes. Succinate dehydrogenase (SDH) is a key mitochondrial enzyme with an important function in the tricarboxylic acid cycle and electron transport chain. SDH deficiency in β-cells has been shown to impair glucose-induced oxidative phosphorylation and disrupt mitochondrial membrane potential, thereby impairing glucose-stimulated ATP production, insulin secretion, and β-cell growth [18]. It has been shown that loss of SDH causes excessive succinate accumulation, resulting in increased lipid synthesis [18]. The role of the SDHAF4 gene in metabolic processes, especially in carbohydrate and lipid metabolism, is not known. Our results indicate that the SDHAF4 gene polymorphism is not a factor affecting the onset of GDM. We only showed an association between this polymorphism and body mass before pregnancy and body mass at birth. Recent studies have indicated that SDHAF4 is involved in many metabolic processes in the liver [31] and SDHAF4 has been shown to affect weight loss in mice. In addition, the suppression of hepatic SDHAF4 was associated with systemic improvement of insulin sensitivity, which may affect patients’ BMI values [32].

MACF1, also known as actin crosslinking family 7 (ACF7), is a spectraplakin present in many tissues and organs involved in numerous cellular processes, playing a key role in cell signaling. MACF1 plays a key role in regulating cell migration and proliferation and maintaining tissue integrity [19]. It also plays a key function in embryogenesis and the development of many tissues and organs.

It has been shown that the MACF1 gene is associated with metabolic syndrome and inflammation [12]. Previous studies have indicated that metabolic syndrome and inflammation are also factors that increase the risk of developing GDM [3]. In our study, there was no significant association between the MACF1 gene rs2296172 A>G polymorphism and GDM risk and clinical parameters in pregnant women.

Our results suggest no association between the studied gene polymorphisms and the risk of GDM, as well as selected clinical parameters. GDM is a metabolic complication occurring in pregnant women that is caused by many factors. The influence of both environmental and genetic factors is considered in this disease. Underlying this disease are both pancreatic islet β cell dysfunction and existing insulin resistance. These processes are influenced by a number of pro-inflammatory factors, as well as disrupting carbohydrate metabolism. The genes we studied mainly affect the processes of carbohydrate metabolism. It seems that the effect of single polymorphisms on the risk of GDM is very small. Due to the complexity of the pathogenesis of this disease, it must be taken into account, along with other environmental factors that increase the risk of developing GDM. It is not excluded that the gene polymorphisms we studied may affect the risk of GDM in other populations. A complete understanding of the role of THADA rs7578597 T>C, SDHAF4 rs1048886 A>G, and MACF1 rs2296172 A>G gene polymorphisms in the pathogenesis of GDM requires multicenter studies in different populations.

## 5. Conclusions

The results of our study suggest that the *THADA* rs7578597 T>C, *SDHAF4* rs1048886 A>G, and *MACF1* rs2296172 A>G gene polymorphisms are not significant factors associated with GDM onset. *SDHAF4* rs1048886 A>G may be associated with body mass before pregnancy and body mass at birth in pregnant women.

## Figures and Tables

**Table 1:** Distribution of *THADA*, *SDHAF4*, and *MACF1* genotypes and alleles in women with GDM and the control group [21,22,23].

	Control Group		GDM		*p* Value ^^^		OR (95% CI)	*p* Value ^^^
	n	%	n	%				
*THADA* rs7578597 genotype								
TT	289	83.05%	229	84.19%	0.89	CC+TC vs. TT	0.92 (0.60–1.41)	0.70
TC	57	16.38%	42	15.44%		CC vs. TC+TT	0.64 (0.06–7.08)	0.71
CC	2	0.57%	1	0.37%		CC vs. TT	0.63 (0.06–7.00)	0.71
						TC vs. TT	0.93 (0.60–1.44)	0.74
						CC vs. TC	0.68 (0.06–7.73)	0.75
Allele								
T	635	91.24%	500	91.91%		C vs. T	0.92 (0.61–1.37)	0.67
C	61	8.76%	44	8.09%				
*SDHAF4* rs1048886 genotype								
AA	222	63.79%	169	62.13%	0.82	GG+AG vs. AA	1.07 (0.77–1.49)	0.67
AG	108	31.03%	86	31.62%		GG vs. AG+AA	1.22 (0.62–2.42)	0.56
GG	18	5.17%	17	6.25%		GG vs. AA	1.24 (0.62–2.48)	0.54
						AG vs. AA	1.05 (0.74–1.48)	0.80
						GG vs. AG	1.19 (0.58–2.44)	0.64
Allele								
A	552	79.31%	424	77.94%		G vs. A	1.08 (0.83–1.43)	0.56
G	144	20.69%	120	22.06%				
*MACF1* rs2296172 genotype								
AA	217	62.25%	163	60.00%	0.85	GG+AG vs. AA	1.10 (0.79–1.52)	0.57
AG	114	32.85%	95	34.81%		GG vs. AG+AA	1.06 (0.51–2.19)	0.87
GG	17	4.90%	14	5.19%		GG vs. AA	1.10 (0.53–2.29)	0.80
						AG vs. AA	1.10 (0.78–1.55)	0.59
						GG vs. AG	1.00 (0.47–2.13)	1.00
Allele								
A	548	78.74%	421	77.39%		G vs. A	1.08 (0.82–1.41)	0.59
G	148	21.26%	123	22.61%				

^ χ^2^ test HWE: control group *p* = 1.00, GDM group *p* = 1.00 for *THADA* rs7578597. HWE: control group *p* = 0.34, GDM group *p* = 0.22 for *SDHAF4* rs1048886. HWE: control group *p* = 0.75, GDM group *p* = 1.00 for *MACF1* rs2296172.

**Table 2:** Clinical parameters of women with GDM stratified according to *THADA* rs7578597 genotype [21,22,23].

Parameters	*THADA* rs7578597 Genotype		
	TT	TC+CC	TT vs. TC+CC
	Mean ± SD		*p* ^&^
Body mass before pregnancy (kg)	73.2 ± 17.3	73.2 ± 14.2	0.82
Body mass at birth (kg)	84.7 ± 15.8	85.6 ± 14.0	0.62
Body mass increase during pregnancy (kg)	11.5 ± 7.2	12.5 ± 6.0	0.40
BMI before pregnancy (kg/m^2^)	26.6 ± 6.1	26.1 ± 5.0	0.74
BMI at birth (kg/m^2^)	30.9 ± 5.7	30.6 ± 4.5	0.88
BMI increase during pregnancy (kg/m^2^)	4.2 ± 2.7	4.4 ± 2.1	0.61
Fasting glucose (mg/dL)	92.8 ± 12.4	90.2 ± 7.2	0.14
Daily insulin requirement (unit)	17.7 ± 30.1	16.6 ± 24.5	0.87
Newborn body mass (g)	3262 ± 565	3360 ± 555	0.29
APGAR (0–10)	9.3 ± 1.0	9.1 ± 1.2	0.43

**^&^** U Mann–Whitney test.

**Table 3:** Clinical parameters of women with GDM stratified according to *SDHAF4* rs1048886 genotype [21,22,23].

Parameters	*SDHAF4* rs1048886 Genotype					
	AA	AG	GG	AA vs. AG	AA vs. GG	AG vs. GG
	Mean ± SD			*p* ^&^		
Body mass before pregnancy (kg)	74.8 ± 17.4	69.7 ± 15.2	76.7 ± 17.9	0.03	0.73	0.16
Body mass at birth (kg)	86.5 ± 16.0	81.1 ± 14.1	89.0 ± 14.9	0.02	0.55	0.10
Body mass increase during pregnancy (kg)	11.7 ± 7.3	11.3 ± 6.8	12.3 ± 6.2	0.85	0.69	0.55
BMI before pregnancy (kg/m^2^)	26.9 ± 6.0	25.7 ± 5.7	27.9 ± 6.3	0.11	0.60	0.17
BMI at birth (kg/m^2^)	31.2 ± 5.5	29.9 ± 5.4	32.4 ± 5.4	0.07	0.38	0.07
BMI increase during pregnancy (kg/m^2^)	4.2 ± 2.7	4.2 ± 2.4	4.5 ± 2.3	0.89	0.67	0.63
Fasting glucose (mg/dL)	91.4 ± 10.3	93.5 ± 14.1	96.0 ± 10.7	0.69	0.16	0.23
Daily insulin requirement (unit)	19.1 ± 32.8	14.9 ± 19.8	15.8 ± 30.2	0.47	0.30	0.50
Newborn body mass (g)	3278 ± 575	3244 ± 536	3420 ± 575	0.52	0.11	0.09
APGAR (0–10)	9.2 ± 1.2	9.3 ± 0.9	9.6 ± 0.6	0.99	0.28	0.26

**^&^** U Mann–Whitney test.

**Table 4:** Clinical parameters of women with GDM stratified according to *MACF1* rs2296172 genotype [21,22,23].

Parameters	*MACF1* rs2296172 Genotype					
	AA	AG	GG	AA vs. AG	AA vs. GG	AG vs. GG
	Mean ± SD			*p* ^&^		
Body mass before pregnancy (kg)	73.0 ± 16.1	74.5 ± 18.5	70.4 ± 16.0	0.69	0.55	0.52
Body mass at birth (kg)	85.3 ± 15.5	85.0 ± 16.2	81.2 ± 13.0	0.96	0.36	0.38
Body mass increase during pregnancy (kg)	12.3 ± 7.2	10.5 ± 6.8	10.8 ± 6.4	0.06	0.63	0.82
BMI before pregnancy (kg/m^2^)	26.4 ± 5.7	27.1 ± 6.4	26.0 ± 5.2	0.45	0.95	0.67
BMI at birth (kg/m^2^)	30.9 ± 5.6	31.0 ± 5.7	30.0 ± 3.5	0.84	0.71	0.55
BMI increase during pregnancy (kg/m^2^)	4.5 ± 2.6	3.9 ± 2.5	4.0 ± 2.4	0.08	0.76	0.70
Fasting glucose (mg/dL)	92.0 ± 11.4	92.8 ± 12.8	91.5 ± 6.1	0.65	0.99	0.80
Daily insulin requirement (unit)	18.9 ± 33.2	16.3 ± 23.3	11.1 ± 10.8	0.54	0.78	0.93
Newborn body mass (g)	3298 ± 529	3251 ± 591	3212 ± 767	0.68	0.94	0.91
APGAR (0–10)	9.2 ± 1.1	9.3 ± 1.1	9.3 ± 1.1	0.83	0.69	0.77

**^&^** U Mann–Whitney test.

**Table 5:** Correlations between *THADA* expression in the placenta and clinical parameters in the GDM group [21,22,23].

Parameters Correlated with Placental Expression of *THADA*	Rs	*p*
Age (years)	0.33	0.10
Fasting glucose (mg/dL)	0.05	0.82
Daily insulin requirement (unit)	0.07	0.74
Body mass before pregnancy (kg)	0.16	0.41
Body mass at birth (kg)	0.25	0.21
Body mass increase during pregnancy (kg)	0.29	0.14
BMI before pregnancy (kg/m^2^)	0.13	0.52
BMI at birth (kg/m^2^)	0.16	0.42
BMI increase during pregnancy (kg/m^2^)	0.22	0.26
Newborn body mass (g)	0.04	0.85
APGAR (0–10)	−0.12	0.56

R_s_—Spearman rank correlation coefficient.

**Table 6:** Correlations between *SDHAF4* expression in the placenta and clinical parameters in the GDM group [21,22,23].

Parameters Correlated with Placental Expression of *SDHAF4*	Rs	*p*
Age (years)	0.26	0.18
Fasting glucose (mg/dL)	0.16	0.42
Daily insulin requirement (unit)	0.02	0.91
Body mass before pregnancy (kg)	0.17	0.39
Body mass at birth (kg)	0.22	0.28
Body mass increase during pregnancy (kg)	0.11	0.59
BMI before pregnancy (kg/m^2^)	0.13	0.51
BMI at birth (kg/m^2^)	0.14	0.48
BMI increase during pregnancy (kg/m^2^)	0.06	0.75
Newborn body mass (g)	−0.01	0.97
APGAR (0–10)	0.02	0.91

R_s_—Spearman rank correlation coefficient.

**Table 7:** Correlations between *MACF1* expression in the placenta and clinical parameters in the GDM group [21,22,23].

Parameters Correlated with Placental Expression of *MACF1*	Rs	*p*
Age (years)	−0.06	0.77
Fasting glucose (mg/dL)	0.09	0.66
Daily insulin requirement (unit)	−0.22	0.27
Body mass before pregnancy (kg)	0.10	0.61
Body mass at birth (kg)	0.20	0.31
Body mass increase during pregnancy (kg)	0.18	0.38
BMI before pregnancy (kg/m^2^)	−0.03	0.87
BMI at birth (kg/m^2^)	0.07	0.74
BMI increase during pregnancy (kg/m^2^)	0.11	0.59
Newborn body mass (g)	0.31	0.11
APGAR (0–10)	0.24	0.22

R_s_—Spearman rank correlation coefficient.

**Table 8:** The mRNA levels of studied genes in the placenta between women with corresponding genotypes.

	*THADA* rs7578597 Genotype			*SDHAF4* rs1048886 Genotype			*MACF1* rs2296172 Genotype		
	TT	TC+CC	TT vs. TC+CC	AA	AG+GG	AA vs. AG+GG	AA	AG+GG	AA vs. AG+GG
	Mean ± SD		*p* ^&^	Mean ± SD		*p* ^&^	Mean ± SD		*p* ^&^
Control group	0.0023 ± 0.0023	0.0028 ± 0.0021	0.46	0.050 ± 0.071	0.022 ± 0.0096	0.85	0.00023 ± 0.00029	0.00066 ± 0.0012	0.73
GDM	0.0031 ± 0.0032	0.0038 ± 0.0029	0.59	0.022 ± 0.023	0.026±0.018	0.47	0.0037 ± 0.013	0.00079 ± 0.0023	0.13

*p*^&^ U Mann–Whitney test.

## Data Availability

Not applicable.

## References

[1] Kim C. (2010). Gestational diabetes: Risks, management, and treatment options. Int. J. Womens Health.

[2] Johns E.C., Denison F.C., Norman J.E., Reynolds R.M. (2018). Gestational Diabetes Mellitus: Mechanisms, Treatment, and Complications. Trends Endocrinol. Metab..

[3] Plows J.F., Stanley J.L., Baker P.N., Reynolds C.M., Vickers M.H. (2018). The Pathophysiology of Gestational Diabetes Mellitus. Int. J. Mol. Sci..

[4] Barbour L.A., McCurdy C.E., Hernandez T.L., Kirwan J.P., Catalano P.M., Friedman J.E. (2007). Cellular mechanisms for insulin resistance in normal pregnancy and gestational diabetes. Diabetes Care.

[5] Dias S., Pheiffer C., Abrahams Y., Rheeder P., Adam S. (2018). Molecular Biomarkers for Gestational Diabetes Mellitus. Int. J. Mol. Sci..

[6] Haythorne E., Rohm M., van de Bunt M., Brereton M.F., Tarasov A.I., Blacker T.S., Sachse G., Silva Dos Santos M., Terron Exposito R., Davis S. (2019). Diabetes causes marked inhibition of mitochondrial metabolism in pancreatic β-cells. Nat. Commun..

[7] Sirico A., Dell’Aquila M., Tartaglione L., Moresi S., Farì G., Pitocco D., Arena V., Lanzone A. (2022). PTH-rP and PTH-R1 Expression in Placentas from Pregnancies Complicated by Gestational Diabetes: New Insights into the Pathophysiology of Hyperglycemia in Pregnancy. Diagnostics.

[8] Sirico A., Rossi E.D., Degennaro V.A., Arena V., Rizzi A., Tartaglione L., Di Leo M., Pitocco D., Lanzone A. (2022). Placental diabesity: Placental VEGF and CD31 expression according to pregestational BMI and gestational weight gain in women with gestational diabetes. Arch. Gynecol. Obstet..

[9] Kawai V.K., Levinson R.T., Adefurin A., Kurnik D., Collier S.P., Conway D., Stein C.M. (2017). A genetic risk score that includes common type 2 diabetes risk variants is associated with gestational diabetes. Clin. Endocrinol..

[10] Kanthimathi S., Chidambaram M., Bodhini D., Liju S., Bhavatharini A., Uma R., Anjana R.M., Mohan V., Radha V. (2017). Association of recently identified type 2 diabetes gene variants with Gestational Diabetes in Asian Indian population. Mol. Genet. Genomics.

[11] Lu Y., Day F.R., Gustafsson S., Buchkovich M.L., Na J., Bataille V., Cousminer D.L., Dastani Z., Drong A.W., Esko T. (2016). New loci for body fat percentage reveal link between adiposity and cardiometabolic disease risk. Nat. Commun..

[12] Kraja A.T., Chasman D.I., North K.E., Reiner A.P., Yanek L.R., Kilpeläinen T.O., Smith J.A., Dehghan A., Dupuis J., Johnson A.D. (2014). Pleiotropic genes for metabolic syndrome and inflammation. Mol. Genet. Metab..

[13] Lyssenko V., Groop L., Prasad R.B. (2015). Genetics of Type 2 Diabetes: It Matters from Which Parent We Inherit the Risk. Rev. Diabet. Stud..

[14] Moraru A., Cakan-Akdogan G., Strassburger K., Males M., Mueller S., Jabs M., Muelleder M., Frejno M., Braeckman B.P., Ralser M. (2017). THADA Regulates the Organismal Balance between Energy Storage and Heat Production. Dev. Cell.

[15] Grarup N., Andersen G., Krarup N.T., Albrechtsen A., Schmitz O., Jørgensen T., Borch-Johnsen K., Hansen T., Pedersen O. (2008). Association testing of novel type 2 diabetes risk alleles in the JAZF1, CDC123/CAMK1D, TSPAN8, THADA, ADAMTS9, and NOTCH2 loci with insulin release, insulin sensitivity, and obesity in a population-based sample of 4,516 glucose-tolerant middle-aged Danes. Diabetes.

[16] Tian Y., Li J., Su S., Cao Y., Wang Z., Zhao S., Zhao H. (2020). PCOS-GWAS Susceptibility Variants in THADA, INSR, TOX3, and DENND1A Are Associated with Metabolic Syndrome or Insulin Resistance in Women With PCOS. Front. Endocrinol..

[17] Settas N., Faucz F.R., Stratakis C.A. (2018). Succinate dehydrogenase (SDH) deficiency, Carney triad and the epigenome. Mol. Cell. Endocrinol..

[18] Lee S., Xu H., Van Vleck A., Mawla A.M., Li A.M., Ye J., Huising M.O., Annes J.P. (2022). β-Cell Succinate Dehydrogenase Deficiency Triggers Metabolic Dysfunction and Insulinopenic Diabetes. Diabetes.

[19] Hu L., Xiao Y., Xiong Z., Zhao F., Yin C., Zhang Y., Su P., Li D., Chen Z., Ma X. (2017). MACF1, versatility in tissue-specific function and in human disease. Semin. Cell Dev. Biol..

[20] Metzger B.E., Gabbe S.G., Persson B., Buchanan T.A., Catalano P.A., Damm P., Dyer A.R., Leiva A.D., Hod M., International Association of Diabetes and Pregnancy Study Groups Consensus Panel (2010). International association of diabetes and pregnancy study groups recommendations on the diagnosis and classification of hyperglycemia in pregnancy. Diabetes Care.

[21] Majcher S., Ustianowski P., Malinowski D., Czerewaty M., Tarnowski M., Safranow K., Dziedziejko V., Pawlik A. (2022). *KCNJ11* and *KCNQ1* Gene Polymorphisms and Placental Expression in Women with Gestational Diabetes Mellitus. Genes.

[22] Ustianowski P., Malinowski D., Kopytko P., Czerewaty M., Tarnowski M., Dziedziejko V., Safranow K., Pawlik A. (2021). *ADCY5*, *CAPN10* and *JAZF1* Gene Polymorphisms and Placental Expression in Women with Gestational Diabetes. Life.

[23] Ustianowski P., Malinowski D., Czerewaty M., Safranow K., Tarnowski M., Dziedziejko V., Pawlik A. (2022). *COBLL1* and *IRS1* Gene Polymorphisms and Placental Expression in Women with Gestational Diabetes. Biomedicines.

[24] Hu C., Zhang R., Wang C., Wang J., Ma X., Lu J., Qin W., Hou X., Wang C., Bao Y. (2009). PPARG, KCNJ11, CDKAL1, CDKN2A-CDKN2B, IDE-KIF11-HHEX, IGF2BP2 and SLC30A8 are associated with type 2 diabetes in a Chinese population. PLoS ONE.

[25] Staiger H., Machicao F., Kantartzis K., Schäfer S.A., Kirchhoff K., Guthoff M., Silbernagel G., Stefan N., Fritsche A., Häring H.U. (2008). Novel meta-analysis-derived type 2 diabetes risk loci do not determine prediabetic phenotypes. PLoS ONE.

[26] Schleinitz D., Tönjes A., Böttcher Y., Dietrich K., Enigk B., Koriath M., Scholz G.H., Blüher M., Zeggini E., McCarthy M.I. (2010). Lack of significant effects of the type 2 diabetes susceptibility loci JAZF1, CDC123/CAMK1D, NOTCH2, ADAMTS9, THADA, and TSPAN8/LGR5 on diabetes and quantitative metabolic traits. Horm. Metab. Res..

[27] Gupta V., Vinay D.G., Rafiq S., Kranthikumar M.V., Janipalli C.S., Giambartolomei C., Evans D.M., Mani K.R., Sandeep M.N., Taylor A.E. (2012). Association analysis of 31 common polymorphisms with type 2 diabetes and its related traits in Indian sib pairs. Diabetologia.

[28] DeMenna J., Puppala S., Chittoor G., Schneider J., Kim J.Y., Shaibi G.Q., Mandarino L.J., Duggirala R., Coletta D.K. (2014). Association of common genetic variants with diabetes and metabolic syndrome related traits in the Arizona Insulin Resistance registry: A focus on Mexican American families in the Southwest. Hum. Hered..

[29] Stuebe A.M., Lyon H., Herring A.H., Ghosh J., Wise A., North K.E., Siega-Riz A.M. (2010). Obesity and diabetes genetic variants associated with gestational weight gain. Am. J. Obstet. Gynecol..

[30] Van Vranken J.G., Bricker D.K., Dephoure N., Gygi S.P., Cox J.E., Thummel C.S., Rutter J. (2014). SDHAF4 promotes mitochondrial succinate dehydrogenase activity and prevents neurodegeneration. Cell Metab..

[31] Wang X., Zhang X., Cao K., Zeng M., Fu X., Zheng A., Zhang F., Gao F., Zou X., Lim H. (2022). Cardiac disruption of SDHAF4-mediated mitochondrial complex II assembly promotes dilated cardiomyopathy. Nat. Commun..

[32] Wang X., Lv W., Xu J., Zheng A., Zeng M., Cao K., Wang X., Cui Y., Li H., Yang M. (2022). Hepatic Suppression of Mitochondrial Complex II Assembly Drives Systemic Metabolic Benefits. Adv. Sci..

